# Optimization, In Vitro, and In Silico Characterization of Theophylline Inhalable Powder Using Raffinose-Amino Acid Combination as Fine Co-Spray-Dried Carriers

**DOI:** 10.3390/pharmaceutics17040466

**Published:** 2025-04-03

**Authors:** Petra Party, Lomass Soliman, Attila Nagy, Árpád Farkas, Rita Ambrus

**Affiliations:** 1 Faculty of Pharmacy, Institute of Pharmaceutical Technology and Regulatory Affairs, University of Szeged, Eötvös Utca 6, 6720 Szeged, Hungary; party.petra@szte.hu (P.P.); lomass.a.soliman@gmail.com (L.S.); 2 Department of Applied and Nonlinear Optics, HUN-REN Wigner Research Centre for Physics, Konkoly-Thege Miklós St. 29-33, 1121 Budapest, Hungary; nagy.attila@wigner.hun-ren.hu; 3 HUN-REN Centre for Energy Research, Institute for Energy Security and Environmental Safety, Konkoly-Thege Miklós St. 29-33, 1121 Budapest, Hungary; farkas.arpad@ek.hun-ren.hu

**Keywords:** dry powder inhaler, spray-drying, theophylline, raffinose, leucine, glycine, aerodynamic particle counter, Andersen cascade impactor, stochastic lung model

## Abstract

**Background/Objectives**: Dry powder inhalation is an attractive research area for development. Therefore, this work aimed to develop inhalable co-spray-dried theophylline (TN) microparticles, utilizing raffinose-amino acid fine carriers intended for asthma therapy. The study addressed enhancing TN’s physicochemical and aerodynamic properties to ensure efficient lung deposition. **Methods**: The process involves spray-drying each formulation’s solution using a mini spray drier. A rigorous assessment was conducted on particle size distribution, structural and thermal analysis, morphology study, in vitro and in silico aerodynamic investigation, and aerodynamic particle counter in addition to the solubility, in vitro dissolution, and diffusion of TN. **Results**: The carriers containing leucine and glycine revealed superior characteristics (mass median aerodynamic diameter (MMAD): 4.6–5 µm, fine particle fraction (FPF): 30.6–35.1%, and amorphous spherical structure) as candidates for further development of TN-DPIs, while arginine was excluded due to intensive aggregation and hygroscopicity, which led to poor aerodynamic performance. TN co-spray-dried samples demonstrated fine micronized particles (D [0.5]: 3.99–5.96 µm) with predominantly amorphous structure (crystallinity index: 24.1–45.2%) and significant solubility enhancement (~19-fold). Formulations containing leucine and leucine-glycine revealed the highest FPF (45.7–47.8%) and in silico lung deposition (39.3–40.1%), rapid in vitro drug release (~100% within 10 min), and improved in vitro diffusion (2.29–2.43-fold), respectively. Moreover, the aerodynamic counter confirmed the development of fine microparticles (mean number particle size = 2.3–2.02 µm). **Conclusions**: This innovative formulation possesses enhanced physicochemical, morphological, and aerodynamic characteristics of low-dose TN for local asthma treatment and could be applied as a promising carrier for dry powder inhaler development.

## 1. Introduction

Therapeutic delivery through the respiratory system has emerged as a promising alternative administration route to treat various diseases by applying effective doses with minimized adverse effects, especially asthma, chronic obstructive pulmonary disease (COPD), cystic fibrosis, and infectious pulmonary diseases such as pneumonia and tuberculosis [1,2,3,4,5,6], with the potential to use drug combinations [7] and high-dose drugs [8,9,10]. Moreover, the pulmonary drug delivery system (PDDS) is now an accepted strategy for delivering vaccines and biological compounds [11,12].

Asthma is a chronic respiratory disease characterized by inflammation and bronchoconstriction [13]. However, local pulmonary therapy for asthma offers significant benefits, reducing the required doses, minimizing the systemic side effects, and concentrating the therapeutic effect on the airways, leading to enhanced symptom control and patient life quality [14]. Various classes of bronchodilators have been considered crucial therapeutic agents for treating asthma [15]. Theophylline (TN), a methylxanthine drug that reduces bronchoconstriction in asthmatic patients, has been administered orally and intravenously, but with notable drawbacks, including serious side effects, drug interactions, and a narrow safety index, which diminished its systemic usage due to the availability of more effective options [16].

However, inhaled TN is reported to be effective in improving the lungs’ function, having anti-inflammatory and potential steroid-sparing efficacy [17,18]. Therefore, low-dose TN is a promising asthma treatment offering anti-inflammatory effects and may serve as an alternative or adjunct to inhaled corticosteroids, particularly for mild-to-moderate cases. It reduces side effects, potentially reverses steroid resistance; however, further studies are required to explore its combined use with inhaled steroids in chronic asthma management [19,20,21].

Over the past few decades, inhalation devices and formulation techniques have emerged to deliver inhalable drugs effectively [22]. Several types of inhalation devices were developed to deliver medications to the lungs. The diversity of these devices is crucial to fit the formulation limitations, therapeutic considerations, and patients’ preferences, including pressurized metered dose inhalers (pMDIs), advanced pressurized metered-dose inhalers with spacers or valved holding chambers, dry powder inhalers (DPIs), soft mist inhalers (SMIs), and nebulizers [23]. The formulation of novel fine carrier-based DPIs is the focus of this work; the successful delivery of medications through the inhalable dry powder technique depends on the device design, pharmaceutical formulation, and patient inhalation force. Consequently, significant efforts have been allocated to optimize the formulation of DPIs of existing and new drugs for both localized and systemic therapeutic effects, using either carrier-free or carrier-based dry powder formulations [24,25].

Lactose has been extensively used as a DPI inert carrier in marketed powder inhalers due to multiple attractive features regarding safety, compatibility, and stability [26]. However, alternative carriers are being studied because of lactose’s limitations concerning the hygroscopic nature and reducing chemical function, which could lead to stability and incompatibility obstacles and restrict its use [27]. Thus, substitute saccharides and amino acids are potential candidates to enhance the stability, dispersibility, and flowability of DPIs [28]. Spray freeze-dried raffinose and cyclodextrin showed advantageous aerodynamic performance [29]. Furthermore, mannitol has emerged as a potential engineered DPI carrier [30], and the mannitol combinations demonstrated a beneficial effect on the aerosolization of multiple developed DPIs [10,31,32,33]. These outcomes highlight the potential of alternative sugars to improve DPI formulation and PDDS efficacy. Therefore, the applied saccharide in this work is raffinose, a non-reducing trisaccharide. A few previous studies investigated its applicability as a DPI carrier. Raffinose has been incorporated into spray-dried microparticles for inhalation [34], exhibiting successful enhanced aerosolization and suitability for protein delivery [35].

Moreover, amino acids, endogenous compatible biomaterials, were found to improve DPIs’ performance by enhancing powder dispersion, moisture protection, and stabilizing the encapsulated medications [36]. However, selecting a specific amino acid could affect the particle’s morphology, surface composition, and interparticle interactions in spray-dried powders [37]. Small-molecule amino acids were investigated as stabilizers and filling agents in DPIs. For instance, l-leucine was intensively studied as an inert carrier in DPIs; it presents aerosolization, dispersibility, and long-term stability enhancement characteristics due to its surface cohesion-reducing effect [37,38,39]; and clinical trials proved its low-risk profile when used as a DPI excipient [40,41]. Furthermore, arginine has been assessed as a therapeutic inhalable powder for tuberculosis and cystic fibrosis [42] and a microparticle-formation enhancer and stabilizer in a nasally inhalable powder, providing homogeneous distribution in the entire nasal cavity [43]. Another study found that the aerosolization of DPIs containing gene vectors can be enhanced by adding multiple amino acids [44]. Despite these potential benefits, a comprehensive investigation into their pulmonary toxicity remains necessary [36].

Eventually, these formulation developments underscore the importance of ongoing research into optimizing PDDSs to enhance treatment efficacy and patient compliance. Multiple previous studies on engineering TN inhalable powder were published using mannitol [45], magnesium stearate [46], and a combination with budesonide to overcome its resistance [47]. However, fine particle design could be beneficial to enhance the pulmonary delivery of a drug with a narrow therapeutic index by reducing the required dose, and TN is an example [48]. Therefore, this experimental work aimed to develop an optimized raffinose-amino acid(s) fine carrier-based DPI formulation of TN for pulmonary delivery, utilizing l-arginine, l-leucine, and glycine. Technically, co-spray-drying using a mini spray drier was applied as a preparation procedure. This manuscript describes a novel formulation of potential low-dose TN inhalable dry powder for local pulmonary delivery for the treatment of asthma, with comprehensive physicochemical, morphological, and aerodynamic assessments. Hence, the presented findings open new potential for future research and development of combined fine carriers for DPI optimization.

## 2. Materials and Methods

### 2.1. Materials

**Figure 1:**
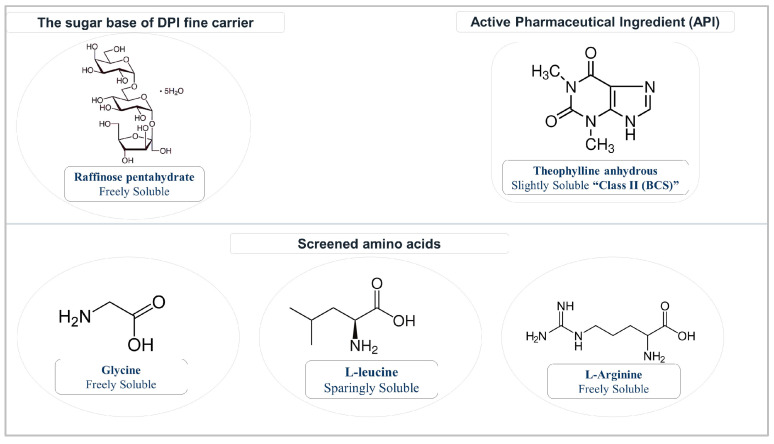
Chemical structure and solubility of the utilized raw API and excipients.

The bronchodilator drug, theophylline anhydrous (TN), was obtained from (Hungaropharma Ltd. Budapest, Hungary). Raffinose pentahydrate (Rf) was used as the main DPI carrier component, obtained from Tokyo Chemical Industry Co., Ltd. (Tokyo, Japan). Additionally, L-leucine (Lc) was purchased from Molar Chemicals Kft. (Budapest, Hungary); L-Arginine (Ar) from Sigma Aldrich Chemie GmbH (Steinheim, Germany); and Glycine (Gl) from VWR International LCC (Leuven, Belgium). Figure 1 illustrates the structure and solubility of the materials utilized. The FTIR test used potassium bromide (J&K Scientific Limited, Beijing, China) for background analysis. Distilled water was collected using an in-house Millipore Direct-Q^®^ 5 UV with a pump water purification unit (Millipore 67,120 Molsheim, France). Hydroxypropyl methylcellulose size-3 capsules (HPMC, Schott Duran, Mainz, Germany) were utilized in the aerodynamic analysis. A mixture of sorbitan monooleate 80 (Span^®^ 80, Sigma Aldrich Chemie GmbH, Steinheim, Germany) and cyclohexane (VWR BDH Chemicals, Paris, France) was applied to form a thin coating layer on the collection plates of the Andersen cascade impactor (ACI). Filtration disks (0.45 μm, Millex-HV syringe-driven filter unit, Millipore Corporation, Bedford, MA, USA) were utilized to filter the aliquots. Simulated lung fluid (SLF) is a simulated bio-relevant fluid that was prepared in-house at pH = 7.4 ± 0.1 [49,50], comprising sodium chloride 0.68 g/L, sodium bicarbonate 2.27 g/L, glycine 0.37 g/L, sodium dihydrogen phosphate 0.1391 g/L, calcium chloride 0.02 g/L, and sulfuric acid (0.1 M) 5.56 mL/L.

### 2.2. Methods

#### 2.2.1. Preparation of Initial Solutions and Spray-Drying Process

**Figure 2:**
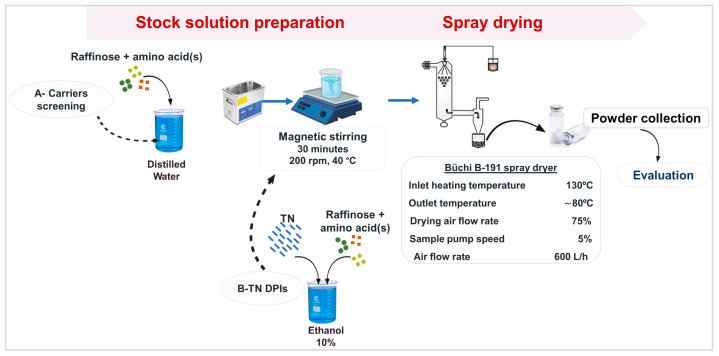
The preparation methods of the Rf-based carrier system (A-Carriers screening) and the TN-DPIs (B-TN DPIs), in addition to the spray-drying operational parameters.

An amount of 200 mL of aqueous stock solutions of each carrier’s components was prepared for further spray-drying, while solutions in ethanol 10% were prepared to produce TN-containing products due to the slight solubility of TN. The solid components were dissolved in the corresponding solvent with ultrasound sonication (Model Elmasonic S30H, Schmidbauer GmbH, Singen, Germany), followed by magnetic stirring with heating (AREC.X, Velp Scientifica Srl, Usmate Velate, Italy) to facilitate the dissolution. Spray-drying was performed to produce the microparticles using a Büchi B-191 mini spray dryer (Büchi, Flawil, Switzerland). Figure 2 reflects the preparation steps and parameters [51,52]. Table 1 demonstrates the detailed composition of each formulation based on our preliminary experiments, which aimed to enhance the yield, load the intended TN dose, and assess the influence of low amino acid content. Afterwards, we modified the composition of TN-Rf-Lc-Gl to TN-Rf-Lc-Gl2 for further investigation. Eventually, the powders were collected after spray-drying in well-sealed glass vials and stored in a desiccator with cobalt crystals at room temperature (23 ± 1 °C) to decrease moisture uptake.

#### 2.2.2. Assessment of TN Solubility

The solubility of the TN was investigated in the raw and spray-dried forms to assess the influence of processing and formulation on the performance. The solubility of TN is expected to be enhanced after spray-drying due to the modification in solid-state, morphological, and surface properties, micronization and the incorporation of Rf and amino acids [53]. The solubility of a drug is of remarkable importance as it affects absorption and bioavailability and, therefore, the drug’s efficiency. The solubility investigation was performed in distilled water and SLF at room temperature (23 ± 2 °C) by adding an excess quantity of each sample to 5 mL of each solvent, followed by using a magnetic heating-plate stirrer (AREC.X, Velp Scientifica Srl, Usmate Velate, Italy) at 400 rpm for 24 h to obtain a saturated drug solution. One milliliter of an aliquot was withdrawn and filtered, and then the TN concentration was analyzed in each saturated aliquot using the UV/Vis Spectrophotometer from ATI-Unicam (Cambridge, UK) at a wavelength of λ = 272 nm. The regression equation was determined as y = 44.014x + 0.0544 in distilled water (R^2^ = 0.981), and y = 60.674x + 0.0111 in SLF (R^2^ = 0.9998), and the calibration curves are available in Figures S1 and S2 of the “Supplementary Materials”, respectively. Three parallel measurements were performed, and the results were presented as an average ± standard deviation.

#### 2.2.3. Structural Analysis by X-Ray Powder Diffraction (XRPD)

To assess the impact of formulation and preparation process on the solid-state and crystal habit, the XRPD diffractograms of unprocessed API, excipients, carriers, and TN co-spray-dried samples were analyzed utilizing a BRUKER D8 Advance X-ray powder diffractometer (Bruker AXS GmbH, Karlsruhe, Germany) equipped with the VNTEC-1 detector (Bruker AXS GmbH, Karlsruhe, Germany). Approximately 10 mg of powder was ground into a fine powder before placing it in the holder. The measurement parameters were set as follows: Cu Kλ1 radiation (λ = 1.5406 Å) served as the radiation source, samples were scanned at 40 kV and 40 mA across an angular range of 3–40° 2-Theta (2θ), with a step period of 0.1 s per step and step size was 0.01°, and calibration of the X-ray apparatus was conducted with standard aluminum oxide powder. Data were processed with the software OriginPro 8.5 (OriginLab Corporation, Northampton, MA, USA) for evaluation, plotting, and adjustment. The obtained diffractograms underwent Kα2 correction, smoothing, and background subtraction before analysis, and the crystallinity index (X_c_%) for each sample was quantitatively determined using Equation (1) on DIFFRAC.EVA.V5.2 software.

X_c_% = (A_crystalline_/A_total_) × 100 (1)

X_c_% is the crystallinity index, “A_crystalline_” refers to the area under the crystalline peaks, and “A_total_” indicates the total area of the diffractogram.

#### 2.2.4. Structural Analysis by Fourier Transform Infrared Spectroscopy (FTIR)

An FTIR spectrometer was utilized to characterize the functional groups in the chemical entities of raw materials, assess any potential interactions among the components of each formulation, and examine the chemical stability of the materials throughout the processing. The FTIR spectra were recorded using Bio-Rad Digilab Division FTS-65A/869 (Philadelphia, PA, USA), covering a wavenumber range from 4000 to 400 cm^−1^ with an optical resolution of 4 cm^−1^. For the analysis, a sample weighing 0.5 mg was mixed with 22 mg of dry potassium bromide in an agate mortar and pestle, and the resulting finely ground mixture was compressed to create a thin transparent disc under a pressure of 10 tons. Each disc was scanned 128 times to improve the signal-to-noise ratio, set at a resolution of 2 cm^−1^ across the specified wavenumber range. Eventually, data analysis, including smoothing, baseline correction, and normalization, was performed using OriginPro 8.5 software (OriginLab Corporation, Northampton, MA, USA).

#### 2.2.5. Thermal Analysis by Differential Scanning Calorimetry (DSC) and Thermogravimetric Analysis (TGA)

The crystallinity and thermal stability of the prepared samples were analyzed using the Mettler Toledo DSC 821e instrument (Mettler Inc., Schwerzenbach, Switzerland). Approximately 3–5 mg of each sample was accurately weighed and placed in an airtight aluminum pan for assessment. The samples were subjected to a heating range of 25 to 300 °C at a constant rate of 5 °C/min while being continuously purged with argon gas at a flow rate of 10 L/h, with an empty aluminum pan serving as a reference. Instrument calibration was performed following the manufacturer’s guidelines, utilizing indium standards. Data analysis was executed with “STARe SW 16.30” software to determine parameters, such as melting point and glass transition temperature (T_g_); OriginPro 8.5 software (OriginLab Corporation, Northampton, MA, USA) was also utilized to visualize the results. Additionally, thermogravimetric analysis (TG-DTA) was conducted using the Mettler Toledo TG 821e system with the STARe thermal analysis program V9.1 (Mettler Inc., Schwerzenbach, Switzerland) under a controlled flow of dry nitrogen gas at 100 mL/min. Samples and reference materials were held in aluminum pans, with scans recorded at a consistent heating rate of 10 °C/min over a temperature range of 30 °C to 300 °C. To minimize moisture interaction, samples weighing approximately 20 mg were quickly weighed after pre-equilibration at room temperature. Weight loss corresponding to moisture evaporation was monitored between 30 °C and 110 °C, and moisture content was calculated based on normalized scan data.

#### 2.2.6. Particle Size Distribution Assessment by Laser Diffraction

Malvern Mastersizer Scirocco 2000 “laser diffraction apparatus” (Malvern Instruments Ltd., Worcestershire, UK) was used to investigate the particle size distribution (PSD) of the prepared samples, employing the dry method by introducing the powders into an air medium. Approximately 100 mg of each product was allocated in the feeder tray for analysis. The dispersion air pressure was calibrated to 2.0 bars to evaluate potential particle attrition. The refractive index for the powders was referenced from the Malvern database, with a measurement duration of 12 s and a vibration feed set to 75%. Each powder was examined in triplicate. The reported parameters for particle size distribution included D (0.1), the median particle size D (0.5), D (0.9), and span values, as defined by Equation (2).

Span = [D(0.9) − D(0.1)]/D(0.5) (2)

D (0.1), D (0.5), and D (0.9) represent the particle size below which 10%, 50%, and 90% of the particles in the sample fall, respectively.

#### 2.2.7. Morphology Study by Scanning Electron Microscopy (SEM)

This investigation evaluated morphology, surface texture characteristics, and approximate dimensions of spray-dried products. Powder samples were analyzed using a scanning electron microscope (SEM) model (Hitachi S4700, Hitachi Scientific Ltd., Tokyo, Japan), operating at 2 kV. To improve the surface conductivity of the powders, samples underwent a gold–palladium coating for 90 s in an argon atmosphere under a high vacuum, facilitated by a sputter coater (Bio-Rad SC 502, VG Microtech, Uckfield, UK). The experimental conditions encompassed an air pressure range of 1.3 to 13.0 mPa, with an accelerating voltage of 10 kV and a beam current of 10 nA. The resultant SEM images were analyzed using ImageJ 1.53t software (https://imagej.net/ij/index.html, accessed on 15 October 2024) to estimate the particle size.

#### 2.2.8. Rheology Study

The flowability characteristics of the prepared powders were evaluated using specific indicators. A density tester (Engelsmann Stampf volumeter, STAV 2003, Ludwigshafen, Germany) was utilized to measure the bulk density and tapped density. Each sample was precisely weighed and transferred into a 5 mL tared graduated cylinder, and its volume was measured before loading it into the density tester. This process allowed for the calculation of bulk density (ρ_bulk_), defined as the mass-to-volume ratio without tapping, which includes interstitial voids [54]. On the other hand, tapped density (ρ_tap_) refers to the density obtained after mechanically tapping the graduated cylinder 1250 times. The methodology employed for these measurements is detailed in the European Pharmacopeia [55]. Additionally, two further indicators of powder flowability were derived from Equations (3) and (4).

CI = [(ρ_tap_ − ρ_bulk_)/ρ_tap_] × 100 (3)

HR = ρ_tap_/ρ_bulk_ × 100 (4)

Carr’s index (CI), known as the compressibility index, and the Hausner ratio (HR), which represents the ratio of ρ_tap_ to ρ_bulk_ of the powder [56]. Measurements were conducted in triplicate, and results were expressed as mean ± SD.

#### 2.2.9. Aerodynamic Assessment

##### In Vitro Aerodynamic Assessment by Andersen Cascade Impactor

The aerodynamic properties of carriers were measured by weight variation using Andersen Cascade Impactor (ACI, Apparatus D, Copley Scientific Ltd., Nottingham, UK), which the European Pharmacopoeia authorizes [57], to determine how effectively the formulations in vitro aerosolized. The inhalation flow rate was set to 60 L/min by a high-capacity pump model (HCP5) and critical flow controller model (TPK) (Copley Scientific Ltd., Nottingham, UK). A mass flow meter (model DFM 2000, Copley Scientific Ltd., Nottingham, UK) was utilized to measure the correct flow rate. There was a 4 s breathe-in, and the setup imitates a regular, healthy breathing rhythm with a 4 L inhalation volume. An amount of 20 mg of each carrier powder was delivered using Breezhaler^®^ single-dose devices (Novartis International AG, Basel, Switzerland) using size 3 HPMC capsules (Capsugel, Bornem, Belgium). Breezhaler^®^ is a breath-activated device that administers drugs for asthma and COPD. To simulate the adherent and humid environment in the respiratory pathways, the collecting plates on the stages were coated with a thin layer of Span 80 and cyclohexane 1% + 99% *w*/*w*, respectively, and left to dry to enhance the attachment of the deposited particles. The eight collection plates, the device (Breezhaler^®^ + capsule), the mouthpiece adapter (MA), and the filter were accurately weighed using an analytical balance before and after inhalation. The weight variation of every mentioned part or stage is calculated to investigate the deposited fraction of the carrier. However, for the assessment of TN aerodynamic properties, the same parameters and conditions were applied, replacing the weight variation measurement with UV detection of TN. Initially, a sample equivalent to 10 mg TN was loaded into the capsule. After inhalation, the device, the MA, the USP induction port, the plates corresponding to each stage, and the filter were rinsed with ethanol 10% to collect the deposited fraction of TN. TN was quantified by UV/Vis spectrophotometry after appropriate dilution (ATI-UNICAM UV/VIS Spectrophotometer, Cambridge, UK) at a wavelength of 272 nm. The regression equation was determined as y = 52.849x + 0.097 in ethanol 10% (R^2^ = 0.9964), and the calibration curve is available in Figure S3 of the “Supplementary Materials”. Three replicates were measured for each sample. Further analysis was continued by calculating the emitted fraction (EF%) based on Equation (5), and the results were plotted and statistically analyzed. Additionally, Inhalytix^TM^ 2.0.6. (Copley Scientific Ltd., Nottingham, UK) software was utilized to determine the fine particle fraction (FPF%), which indicates the fine microparticles < 5 µm based on Equation (6), median mass aerodynamic diameter (MMAD), which should fall within 1–5 µm for better deep lung deposition, and geometric standard deviation (GSD), referring to the aerodynamic PSD.

EF% = [(initial mass − final mass remaining in capsule)/(initial mass)] × 100 (5)

FPF% = [(mass of fine particles (<5 μm))/(total emitted mass)] × 100 (6)

##### In Silico Aerodynamic Assessment

The in silico modeling utilized in this study serves as a computational framework to simulate drug deposition patterns within the respiratory tract, encompassing the extrathoracic and pulmonary regions, which include the bronchial and acinar areas. The total exhaled drug quantity was determined by calculating the initial metered amount (100%) and subtracting the portions retained in the lungs, extrathoracic airways, and remaining within the inhaler. This approach offers an ethical alternative to traditional animal testing [58]. A stochastic lung deposition model was employed, with thorough details of the numerical methodology available in the previous literature [59]. This model accurately represented particle movement through a realistically structured asymmetric branching airway system, applying anatomical data. It incorporated numerous particle characteristics, such as size, shape, and density, alongside patient-specific breathing parameters, including inhaled volume, inhalation and exhalation durations, breath-holding (BH), and whether breathing occurred through the nose or mouth. Aerodynamic particle size distributions (APSDs) obtained from ACI measurements informed the inputs for the numerical model. The inhalation parameters specifically reflected those of an asthma patient using a Breezhaler^®^ inhalation device, with an inhaled volume of 4 L and a duration of 4 s, consistent with the current flow rate measurements of 60 L/min. The study also examined the impact of BH post-inhalation by analyzing two different durations: 5 s and 10 s. Previous research has validated the efficacy of this computational deposition model for inhalable pharmaceutical formulations [60,61].

##### Aerodynamic Particle Counter (APC) Characterization

The enhanced formulations underwent an aerodynamic particle size (APS) analysis [62] to assess the PSD and confirm suitability for deep lung delivery. Size-3 HMPC capsules containing the drug product were administered using a Breezhaler^®^ (Novartis International AG, Basel, Switzerland). The experimental setup comprised a breath simulator (mimicking human inhalation), an induction port (simulating the upper respiratory tract), a vacuum pump with a critical flow controller, and an APS. A high-capacity pump (HCP5; Copley Scientific Ltd., Nottingham, UK) and flow controller (TPK 2000; Copley Scientific Ltd., Nottingham, UK) maintained consistent airflow. The pump compensated for the airflow consumed by the APS and system losses. The initial airflow rate was determined by measuring flow through the inhaler’s mixing unit upper inlet under standard conditions. During testing, the breath simulator generated a defined flow profile activating the DPI via the mixing inlet (Copley Scientific Ltd.). This inlet interfaced the DPI-activating airflow with the mainstream conveying aerosolized particles to the APS. An isokinetic nozzle delivered the mainstream to the APS (TSI 3321; TSI Incorporated, Shoreview, MN, USA). The APS analyzed the aerosol, providing PSD data across 52 channels (0.5–20 μm aerodynamic diameter). Particle size was determined via time-of-flight measurements in an accelerated airflow. The APS operated at 1 L/min with a 5 s continuous sampling time, selected based on inhalation profile and particle residence time. The in-house breath simulator produced inhalation/exhalation flows using a PLC-controlled servo motor-driven piston pump. Inhalation volume ranged from 0.1 cm^3^ to 6800 cm^3^, with adjustable time resolution (20, 50, or 100 ms). The inhalation waveform was adapted from published data [63,64], and a flow controller maintained a 60 L/min flow rate (verified using a TSI 4000 thermal mass flow meter; TSI Incorporated, Shoreview, MN, USA; 0.5–200 Nl/min range). Five parallel samples (10 mg of TN equivalent) were analyzed; results are reported as median, mean, geometric mean, mode, and geometric standard deviation (GSD).

#### 2.2.10. In Vitro Dissolution

The in vitro dissolution test could contribute to a better understanding of the bio-pharmaceutical aspects of a DPI, quality control, and the development of novel DPIs. TN release was assessed using an adjusted paddle method (Hanson SR8 Plus, Teledyne Hanson Research, Chatsworth, CA, USA) as indicated by the European Pharmacopeia [65]; the paddle method was found to be the most reliable and reproducible technique [66]. One-hundred-milliliter dissolution vessels with reduced paddle size were utilized, and a volume of 50 mL dissolution medium of fresh synthetic SLF was chosen to simulate the estimated physiological lung fluids [67] and maintain sink conditions. Containers were filled with 50 mL of SLF, and the temperature was adjusted to 37 °C. The parameters were formulated according to the specific conditions of human airways. Then, 10 mg of raw TN and samples equivalent to 10 mg of TN based on drug content analysis were distributed in the medium. The pulmonary dose of TN was determined to be a tenth of its lowest oral dose, which is 100 mg. A 50 rpm-paddles rotation rate was utilized, and the test duration was 60 min. Then, 2 mL of each sample was extracted and replaced with fresh preheated medium after 1, 2, 3, 4, 5, 10, 15, 30, and 60 min. The aliquots were filtered using filtration disks. After filtration, the samples were measured using a spectrophotometer at a wavelength of 272 nm (UV/Vis ATI-UNICAM UV/Vis Spectrophotometer, Cambridge, UK). The measurements were conducted three times for each sample.

#### 2.2.11. In Vitro Diffusion Evaluation

**Figure 3:**
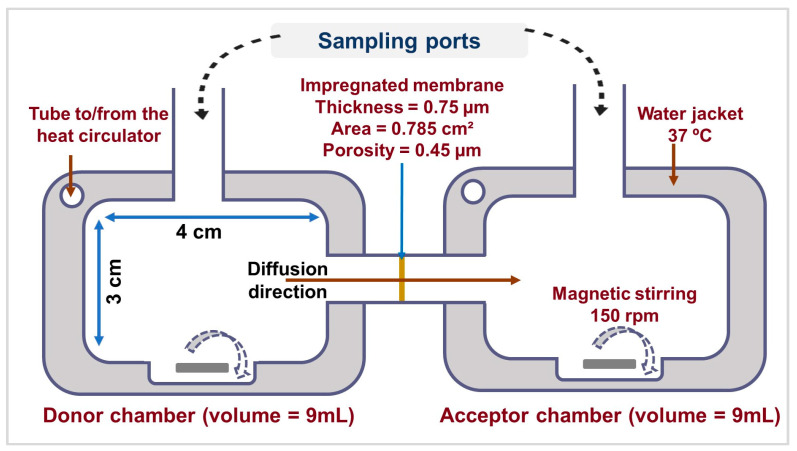
The configuration of the horizontal diffusion cells for in vitro permeation study of TN and the characteristics of various parts are illustrated.

Adapted horizontal 3-D printed diffusion cells were specifically created for unconventional drug delivery routes such as PDDS [68]. Figure 3 illustrates the setup of the developed device and the features of its different parts. The utilized method and parameters were validated by an in vitro–in vivo correlation study [69] and applied by our research group [32,62]. The study was employed to evaluate the in vitro permeability of raw TN and the TN co-spray-dried formulations, which previously exhibited optimal characteristics, from SLF to phosphate buffer. Nine milliliters of SLF served as the “donor” phase, and nine milliliters of phosphate-buffered solution (pH = 7.4) functioned as the “acceptor” phase to simulate the epithelial cell. A circular cellulose filter (RC 55 Whatman^TM^ GE Healthcare Life Sciences, Buckinghamshire, UK) was saturated in a hydrophobic liquid (isopropyl myristate) for 24 h. The excessive liquid was removed, and the membrane was inserted into the donor–acceptor interface. Continual agitation and a regulated temperature were maintained throughout the experiment to mimic the biological conditions. The temperature was adjusted to 37 °C using a water-circulator thermostat. The magnetic stirrer (CS-Smartlab Devices Ltd., Kozarmisleny, Hungary) was set to 150 rpm. Powder samples equivalent to 10 mg of TN were distributed in the donor phase. Aliquots were withdrawn at (5, 10, 15, 30, and 60 min) from the acceptor phase and replaced with fresh preheated medium; the permeated quantity was calculated via UV/Vis spectrophotometer (ATI-UNICAM UV/VIS Spectrophotometer, Cambridge, UK) at a wavelength of 272 nm. The regression equation was determined as y = 59.816x + 0.0055 in phosphate buffer (R^2^ = 0.9941), and the calibration curve is available in Figure S4 of the “Supplementary Materials”. All measurements were performed in triplicate. The flux (J) was determined by dividing the quantity of TN that diffused across the membrane (m) by the membrane’s surface area (A) and the experiment duration (t) [μg/cm^2^/h], as shown in Equation (7). The permeability coefficient (K_p_) [cm/h] is calculated based on Equation (8), which is the ratio of flux (J) and the drug concentration (C_d_) [µg/cm^3^] in the donor phase. The relative permeation at 60 min (RP_60_) was also determined as a ratio of the diffused quantity from the samples to the control (raw TN).

J = (m)/(A × t) (7)

K_p_ = (J)/C_d_ (8)

#### 2.2.12. Statistical Analysis

Statistical analysis was conducted using GraphPad Prism 8.0.1 software (GraphPad Software, San Diego, CA, USA). A one-way analysis of variance ANOVA was performed, and *p*-values < 0.05 were deemed to reflect statistically significant variations. All data presented are expressed as the mean ± standard deviation (SD) from three independent measurements (n = 3).

## 3. Results

In the current study, we developed and optimized the TN combined DPI formulation by co-spray-drying for pulmonary delivery using Rf as a sugar base and amino acids (Lc, Gl) as potential formulation enhancers.

### 3.1. Screening Results of the Carriers

**Figure 4:**
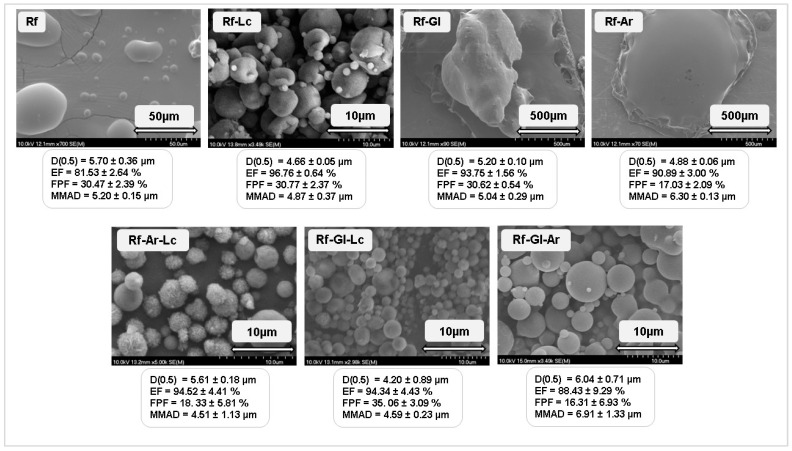
Summary of the results of the screened Rf-based carriers. SEM images, particle size, and aerodynamic properties.

Six batches of TN powder with various compositions were produced. The collected dry powders were evaluated regarding their surface morphology, particle size, physicochemical properties, aerodynamic characteristics, and in vitro release and diffusion. The results of the screening of the prepared Rf-based carriers are illustrated in Figure 4. Structural properties: All spray-dried carriers exhibited a partially amorphous structure with X_c_ ranging between 33.6 and 43.0%, as determined from the XRPD patterns, which revealed lower peak intensities than the raw excipients. Furthermore, the FTIR spectra of carriers indicated minor alterations upon spray-drying, which is expected due to water evaporation from Rf pentahydrate and solid-state transformation from crystalline to an amorphous mixture. Thermal properties: DSC thermograms verified the transition to partially amorphous powders during spray-drying, which lacked sharp endothermic peaks. TGA revealed relatively low residual water content in all carriers, ranging between 1.40% and 1.97%. Rheological properties: All prepared carriers exhibited poor flowability (Hausner ratio: 1.64–2.19, and CI%: 38.95–54.20%) yet low density (bulk density: 0.19–0.51 g/cm^2^, and tapped density: 0.41–0.84 g/cm^2^). However, lower density values are typically considered more efficient for aerosolization; it could also be varied based on the formulation aspects [70,71]. Laser diffraction analysis: It is generally acceptable that D [0.5] < 5 µm is promising for pulmonary application [72]. The D [0.5] of Rf, Rf-Gl, Rf-Ar-Lc, and Rf-Gl-Ar slightly exceeded this limit. However, Rf-Lc, Rf-Ag, and Rf-Gl-Lc obtained the required D [0.5]. Morphological properties: SEM images of the carriers do not demonstrate crystal structure, which correlates with the previous findings. Rf, Rf-Gl, and Rf-Ar showed uninformative SEM results, which could be attributed to particle aggregation due to the hydrophilic properties of the components, particularly Ar, having hygroscopicity properties [73]. The ternary carriers demonstrated a regular spherical shape of microparticles and so did Rf-Lc with a somewhat wrinkled surface. Aerodynamic properties: EF is preferred to fall into the 85–115% range [74]; all carriers fulfilled this criterion except Rf. MMAD of carriers meet the requirement (1–5 µm) approximately; Rf-Ar and Rf-Gl-Ar are exceptions. The FPF of carriers containing Ar is significantly lower than the remaining ones, indicating a smaller fraction of the emitted mass that entered the deep lungs [75]. Aerodynamic performance was mainly considered in carriers’ selection for further development, specifically the FPF. Based on these results, Rf, Rf-Lc, and Rf-Gl as binary combinations, and Rf-Lc-Gl as a ternary combination emerged as promising candidates for TN-DPIs development as fine co-spray-dried powders. Rf was also considered a control formulation that does not contain amino acids to assess the effect of their incorporation. On the other hand, Ar was excluded from the upcoming formulation steps due to its properties, which might adversely influence the integrity and deteriorate the aerodynamic performance of the developed powders. Its hygroscopicity arises from its unique guanidinium group, a highly polar, positively charged side chain. It forms hydrogen bonds with water molecules, enhancing electrostatic attraction to water’s oxygen atoms. These interactions enable arginine to absorb and retain moisture, promoting clustering and aggregation [76].

### 3.2. Solubility of TN

**Figure 5:**
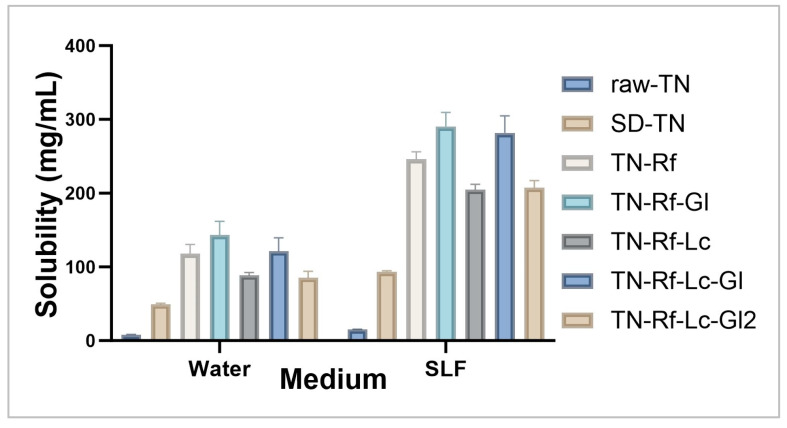
Results of solubility analysis of TN, raw drug vs. spray-dried samples in distilled water and SLF.

The solubility of TN in both distilled water and SLF is presented in Figure 5. Overall, TN’s solubility in SLF (pH = 7.4) is approximately two-fold higher than in water, which could be attributed to the weak base chemical structure, having a dissociation constant pKa of 8.81. SD-TN’s solubility exhibited a remarkable enhancement compared to raw TN (raw TN solubility is 7.36 mg/mL (https://pubchem.ncbi.nlm.nih.gov/ accessed on 29 March 2025)). Notably, TN-Rf-Lc showed less solubility than other formulations that contain additional Gl. On the other hand, TN-Rf-Lc-Gl2 (TN; Rf ratio is 1:2) had less solubility than TN-Rf-Lc-Gl (TN: Rf ratio is 1:3) due to the lower TN embedded in the latter. The solubility metrics for co-spray-dried samples revealed an 11–18-fold increase in TN’s water (raw-TN: 7.93, SD-TN: 49.00, TN-Rf: 118.07, TN-Rf-Gl: 143.38, TN-Rf-Lc: 85.25, TN-Rf-Lc-Gl: 121.29, and TN-Rf-Lc-Gl2: 88.47 mg/mL) and a 13–19-fold increase in SLF (raw-TN: 15.34, SD-TN: 93.41, TN-Rf: 246.28, TN-Rf-Gl: 290.62, TN-Rf-Lc: 207.42, TN-Rf-Lc-Gl: 281.93, and TN-Rf-Lc-Gl2: 204.89 mg/mL) compared to the raw TN’s solubility in the corresponding solvent. This significant enhancement in solubility is mainly due to the increased surface area available for solvent interaction, facilitated by the spray-drying and micronization processes. The water-soluble components Rf and Gl also improved TN’s wettability considerably. The essential mechanisms promoting TN’s solubility enhancement of our samples include the development of solid dispersions with partial amorphous characteristics and the reduction in particle sizes to the microscale [77]. Enhancing the solubility of TN is anticipated to lead to quick drug release in the SLF and improved permeability. Consequently, the results suggest that the employed preparation techniques and excipients were effectively optimized for slightly water-soluble compounds.

### 3.3. X-Ray Diffractograms

**Figure 6:**
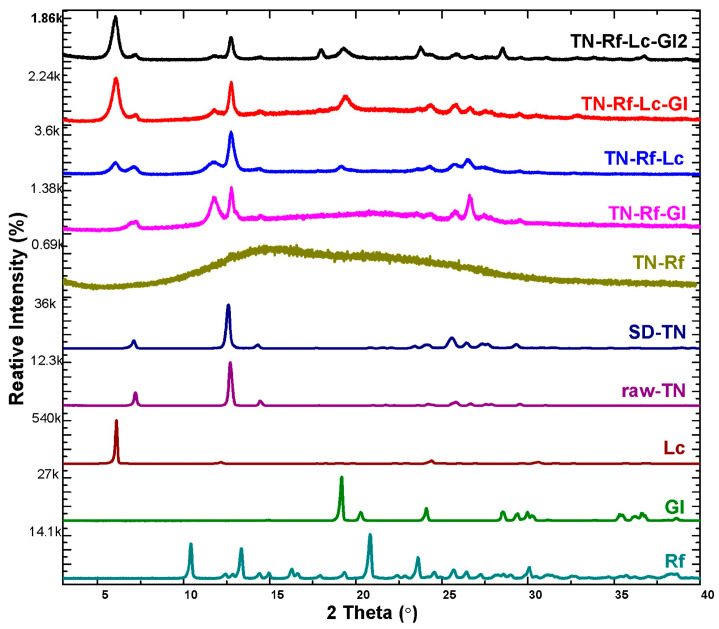
X-ray diffractograms of raw materials and TN spray-dried samples.

The XRPD patterns provide valuable information on the solid state before and after processing, which has a crucial influence on physicochemical and biological performance. The diffractograms of unprocessed TN and excipients demonstrated high-intensity characteristic peaks, indicating a highly crystalline structure, as shown in Figure 6. SD-TN demonstrated no shift or absence of any characteristic peak (at 7.2°, 12.7°, 14.5°, and multiple peaks between 20 and30° 2 theta) compared to the raw-TN. TN characteristic peaks are comparable with a previous study of its solid-state forms [78]. The X_c_ of TN decreased slightly from 94.6% to 88.1% upon spray-drying. On the other hand, there is a profound decrease in the relative intensity of the characteristic peaks of the TN co-spray-dried formulations, as illustrated in Figure 6, indicating a partially amorphous structure after spray-drying. Considering this, the X_c_ values confirm the amorphous structure (TN-Rf: 24.1%, TN-Rf-Gl: 26.2%, TN-Rf-Lc: 42.7%, TN-Rf-Lc-Gl: 36.4%, and TN-Rf-Lc-Gl2: 45.2%). TN-Rf showed the least crystallinity (24.1%) with an amorphous halo (broadband), while the other formulations that contain amino acids exhibited well-detected peaks and higher X_c_. Previous studies found solid-state transformation in the diffractograms of sugars such as Rf [35] and amino acids [79,80], after spray-drying.

### 3.4. FTIR Spectra

**Figure 7:**
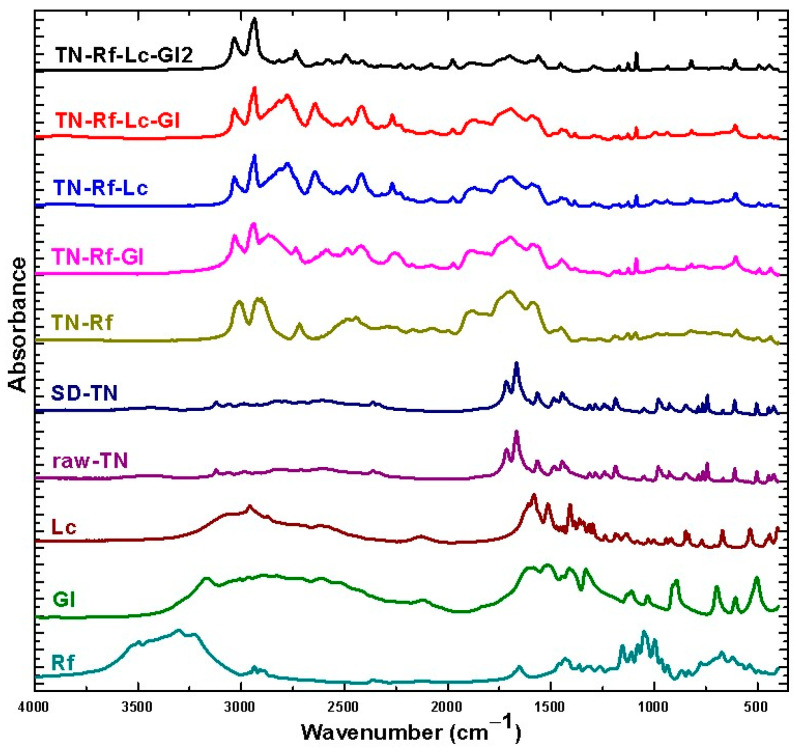
FTIR spectra of raw materials and TN spray-dried samples.

Figure 7 shows the FTIR spectra of the raw materials and TN co-spray-dried samples. The spectra of unprocessed materials correlate highly with the reference spectra (https://spectrabase.com accessed on 20 November 2024). The characteristic IR absorption bands of TN are C=O stretching vibrations of carbonyl groups (1716, 1667 cm^−1^), the N-H stretching band of secondary amine (3121 cm^−1^) [81], C-H (aromatic and aliphatic) stretching vibration multiple peaks (2800–3100 cm^−1^), and overlap between the C=C stretching vibration of the aromatic ring (1567–1445 cm^−1^) and the C-N stretching vibration of the aromatic ring (multiple peaks 1300–1600 cm^−1^). No significant transformation in TN was observed upon drying alone. All TN characteristic peaks were detected in the co-spray dried TN samples, with overlap with other components’ spectra and higher peak intensities between 1500 and 3000 cm^−1^. Additionally, a slight shift to a lower wavenumber in the N-H band peak was detected. Multiple peaks (1500–2000 cm^−1^) in the spectra of co-spray dried samples are approximately corresponding, indicating an interaction between TN and Rf, which could be attributed to van der Waals bonds upon processing. Moreover, the intensity of peaks below 1500 cm^−1^—the fingerprint region—decreased. O-H stretching 3200–3600 cm^−1^ of Rf disappeared in the processed samples due to water evaporation and the transformation into the amorphous form, which changed the FTIR spectrum. A previous study referred to changes in FTIR spectra of amino acids upon spray-drying due to the changes in the structure and molecular interaction with other excipients [82].

### 3.5. Thermograms

**Figure 8:**
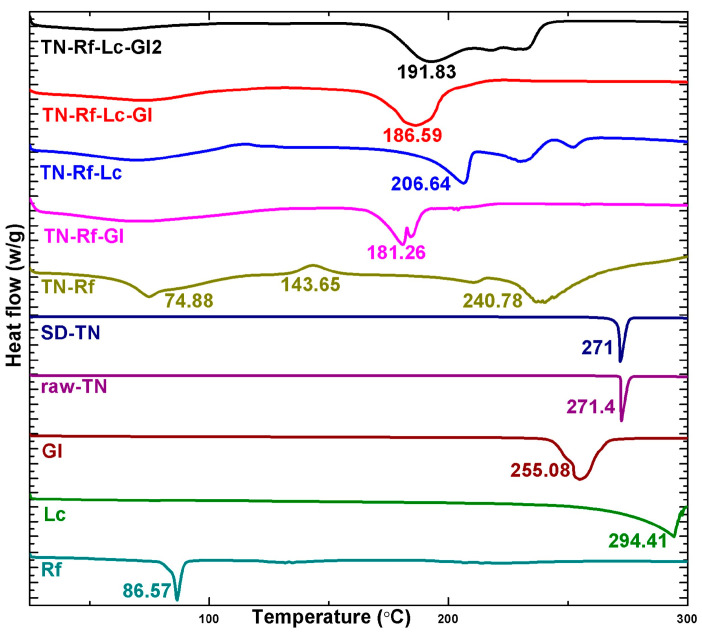
DSC thermograms of raw materials and TN spray-dried samples.

The DSC thermograms of raw materials and TN co-spray-dried samples are presented in Figure 8. This investigation was conducted to test the melting point of the raw materials and the thermal changes after spray-drying, which supports the solid-state results. All raw materials and SD-TN had well-recognized sharp endothermic peaks reflecting highly crystalline structure and melting points as follows: Rf: 86.57 °C, Gl: 255.08 °C, Lc: 294.41 °C, TN: 271.4 °C. The endothermic peak of Rf is called the dehydration peak, which refers to the loss of structural water molecules [83]. No endothermic behavior was noted below 90 °C in the raw-TN thermogram, confirming the anhydrous form [84]. However, the SD-TN thermogram showed a slight shift to a lower melting point (271 °C), which could be due to a change in the crystal forms and reduced particle size. All co-spray-dried products demonstrated broad endothermic peaks, indicating a partially amorphous structure, while the remaining crystals initiated melting at lower temperatures due to the micronization after spray-drying and increased surface area. This phenomenon is referred to as melting point depression. TN-Rf exhibited a slight recrystallization (exothermic peak) at 143.65 °C, while it disappeared in the presence of amino acids. Moreover, the incorporation of amino acids reduced the melting point of TN significantly compared to TN-Rf, SD-TN, and raw TN.

TGA thermograms were analyzed to extract the residual water content, which is revealed by the initial weight loss region ranging between 30 and 110 °C. The detected residual water content in TN co-spray-dried samples ranged between 0.00% and 3.03%. Additionally, raw TN and SD-TN had zero water content due to the hydrophobic nature of TN. However, Lc was found to have the moisture protection effect of dry powders [79,85].

### 3.6. Particle Size Distribution by Laser Diffraction

Spray-drying reduced the median particle size D [0.5] to the fine micro-size range (<6 µm), as exhibited in Table 2. Span values of all spray-dried formulations indicated a relatively narrow size distribution except for TN-Rf-Lc-Gl; the latter showed a wide range of particle size values between D [0.1] and D [0.9]. However, generally lower span values (1–2) are considered more favorable for consistent pulmonary drug delivery, and the span value could be affected by composition, as concluded in a previous study [86]. The obtained D [0.5] falls into the desired 1–5 µm range for respirable fine microparticles, which is an essential factor that influences the aerodynamic efficiency of DPIs in addition to the other powder properties [87]. The micro-engineering is expected to positively affect the drug solubility, drug release rate, and permeability due to the increased surface area available for biological fluids [88]. The measured PSD results are attributed to the preparation process with less effect of formulation differences or particle-particle interactions. However, SD-TN has less tendency to form large particles upon spray-drying; it revealed the best span value and lowest D [0.9], which could indicate that the used excipients induced the formation of larger and more porous particles with less density.

### 3.7. Morphological Characteristics

**Figure 9:**
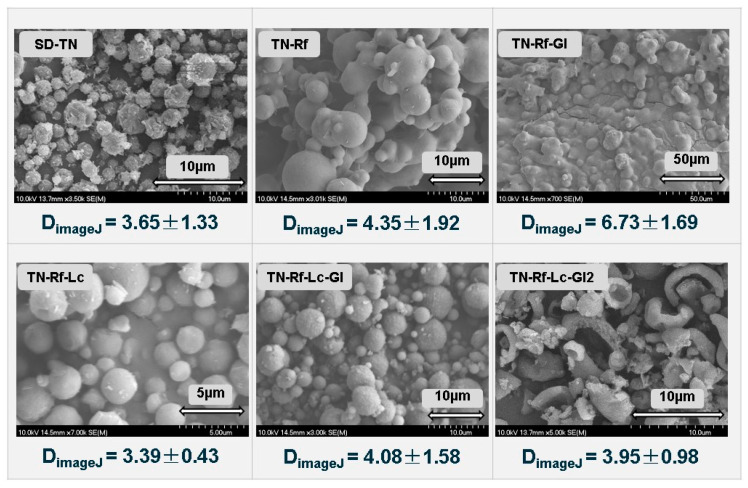
SEM images of TN spray-dried samples and the approximate particle size measured by ImageJ software (D_imageJ_).

The SEM images of all spray-dried formulations are demonstrated in Figure 9. The SD-TN exhibits a well-defined crystalline structure, forming spherical aggregates with rod-like crystals on the rough surface, corresponding with the DSC and XRPD results. It could be attributed to the crystallization of TN within the spray-dried droplets. In contrast, TN-Rf, TN-Rf-Lc, and TN-Rf-Lc-Gl revealed an approximately spherical shape characterized by their remarkably smooth surfaces. However, well-observed aggregation in the case of TN-Rf and TN-Rf-Gl could be attributed to the absence of Lc in both formulations, which could help to form a hydrophobic shell [89]. On the other hand, TN-Rf-Lc-Gl2 exhibited hollowed and fractured spheres, which could be a result of higher TN content than the other ones. The spherical shape was widely considered suitable for pulmonary delivery but less than pollen-shaped ones [90]. The utilized combinations had a profound effect on morphology, alongside the spray-drying forming solid particles and inhibiting the crystallization of Lc and Gl when applied at low concentrations, resulting in a predominantly amorphous structure, as observed by previous studies [91,92]. Nevertheless, these studies found particles with a slightly wrinkled surface. The approximate diameter estimated by ImageJ software proved the formation of microparticles ranging from 2 to 5 µm; TN-Rf-Gl is an exception due to intensive aggregation. It could be concluded that spray-drying is a valuable technique for developing fine, approximately spherical microparticles with partially amorphous properties, which is critical in enhancing the bioavailability of embedded drugs [93].

### 3.8. Density and Flowability Properties

The powder rheological characteristics are demonstrated in Table 3. Unprocessed and spray-dried TN have significantly higher bulk and tapped density than the TN co-spray-dried formulations, which could be attributed to crystal habit. Raw and spray-dried TN showed higher X_c_ than the other formulations, revealing an ordered arrangement of molecules within the solid-state structure, which typically leads to high density compared to the amorphous; the latter lacks the highly regular molecular arrangement [94]. Consequently, the co-spray-dried particles could be large and porous compared to smaller, dense particles of SD-TN as we found in the laser diffraction study. However, it was stated that lower tapped density is advantageous for deep lung drug delivery [95], achieving more reliable flow during the inhalation. It was stated that spray-drying is expected to reduce the powder density [89], which correlates with our findings. These outcomes suggest that powder morphology and structure affect the rheological properties significantly, such as density and flowability [96]. Moreover, the HR and CI of all formulations are approximately comparable, indicating poor to possible flowability.

### 3.9. Aerodynamic Properties

#### 3.9.1. In Vitro Aerodynamic Characterization, Andersen Cascade Impactor (ACI)

**Figure 10:**
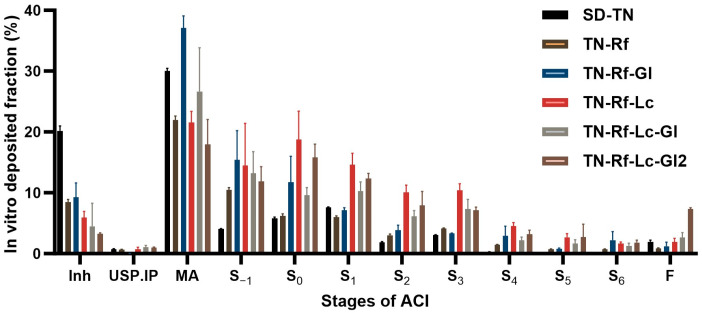
In vitro aerodynamic performance of the developed TN samples, deposition via Andersen cascade impactor (Inh: Breezhaler + capsule; MA: mouthpiece adaptor; IP: induction port; S: stage; F: filter).

The main outcomes of the ACI investigation are EF, FPF, and MMAD in addition to GSD and delivered doses, as shown in Table 4. These findings are extracted from the in vitro deposition results demonstrated in Figure 10 and the Inhalytix^TM^ 2.0.6. software processing. SD-TN recorded the lowest EF, while other formulations had comparable and high ones, falling within the acceptable range (85–115%) [74]. Higher EF values are cost-effective to deliver the given dose. However, FPF is another crucial factor influencing the delivered dose to the real target site because it reflects particles with respirable size (generally 1–5 μm); thus, higher FPF values are also desirable. TN-Rf-Lc and TN-Rf-Lc-Gl2 recorded the best FPFs. MMAD is the aerodynamic particle diameter obtained from the ACI results. TN-Rf-Lc and TN-Rf-Lc-Gl2 showed the most approximate MMAD to the accepted mentioned range, which correlates with the FPF outcomes. However, GSD refers to the distribution of aerodynamic particle size, and the lower the GSD obtained, the more consistent and reliable TN delivery is expected. There is no standard range of accepted GSD, yet it is generally considered satisfactory below 2; however, it depends on the formulation and microparticle properties. Therefore, up to 2.7 could be advantageous [97]. TN-Rf-Lc, TN-Rf-Lc-Gl, and TN-Rf-Lc-Gl2 recorded significantly higher delivered doses. Eventually, by considering the aerodynamic parameters, TN-Rf-Lc and TN-Rf-Lc-Gl2 exhibited the best performance.

#### 3.9.2. In Silico Aerodynamic Characterization

**Figure 11:**
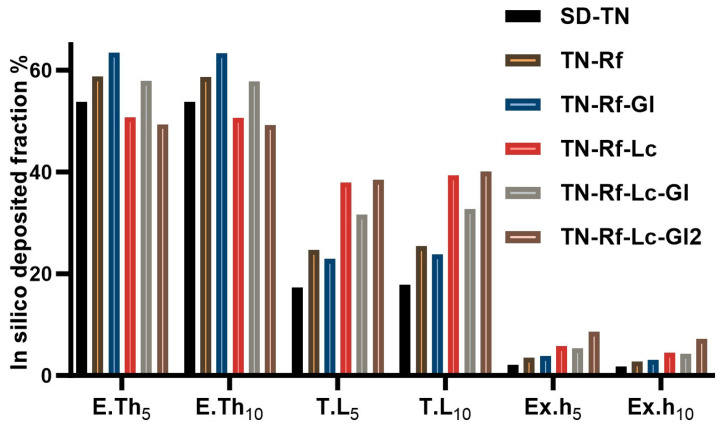
Aerodynamic performance of the developed TN samples. In silico aerodynamic deposition at 5 and 10 s breath hold (E.Th: extrathoracic fraction; T.L: total lung fraction including bronchial and acinar; Ex.h: exhaled fraction).

Computational in silico modeling was used to predict the deposition profile of the TN powders in the respiratory system. The deposited fractions shown in Figure 11 are highly associated with the ACI aerodynamic results. TN-Rf-Lc and TN-Rf-Lc-Gl2 had significantly higher total lung deposition (BH5s: 37.87–38.50%, BH10s: 39.28–40.11%, respectively) than the other formulations (17.31–32.76%). However, no significant difference was found between the general results of each formulation with either 5 s or 10 s BH time. On the other hand, a previous study by our research group found that 10 s BH reduced the exhaled fraction and enhanced the total-lung deposited fraction compared to 5 s BH [98]. Therefore, the patients could prefer a 5 s BH, which improves their compliance. In summary, a relatively low exhaled fraction was detected (1.75–8.63%), which is attributed to the particle size being close to the upper limit as shown in Table 2 and Table 5; therefore, fewer extra-fine particles existed. However, the relatively coarse particles tend to deposit in the upper airways (including nose, mouth, pharynx, and larynx), reflecting the extrathoracic fraction (49.21–63.43%), which is considered high compared to previous studies [32,99,100] due to the higher MMAD values achieved in our experiments. This area is recommended to be addressed in the upcoming work on fine DPIs.

#### 3.9.3. Aerodynamic Particle Counter (APC)

APC was utilized to measure the number, surface, and volume aerodynamic particle size of TN-Rf-Lc and TN-Rf-Lc-Gl2. APC was independent of ACI and provided a more precise breathing pattern. Moreover, it supported the outcomes of the ACI, enhancing the understanding of the behavior of a DPI in the respiratory system. The analysis of formulations TN-Rf-Lc and TN-Rf-Lc-Gl2 revealed the size from the three different perspectives as shown in Table 5, existing within the range of 1.45–5.67 μm, which approximately correlates with the definition of fine particles for effective deep lung delivery, which should range (2.1–5 μm) [101] and the general optimal range (1–5 μm) [87] for reaching the bronchiolar and alveolar region and bypassing the upper airways with a limited exhaled fraction. Moreover, GSD values that reflect the PSD are relatively low, supporting reliable and consistent TN delivery.

### 3.10. In Vitro Drug Release

**Figure 12:**
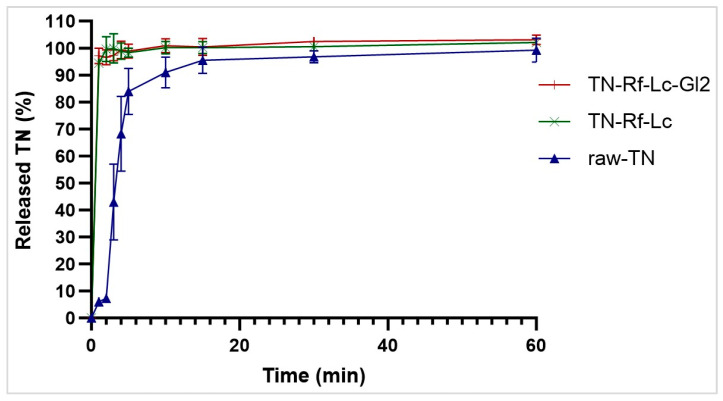
In vitro dissolution profile of raw-TN, TN-Rf-Lc, and TN-Rf-Lc-Gl2 formulations.

The release profile of TN is illustrated in Figure 12. While TN exhibits a slight water solubility, it is well recognized in PDDS that the administered dose is preferred to dissolve and be released rapidly upon deposition to minimize the potential of a clearance mechanism such as macrophage uptake [102]. The lowest TN release rate was observed from the raw drug. On the other hand, a total release of TN from TN-Rf-Lc and TN-Rf-Lc-Gl2 was observed after about 5 min, showing no statistically significant differences, even though the combinations of excipients used varied. Nevertheless, the quick release of TN from the co-spray-dried samples can be attributed to the micronization process and the reduction in crystallinity indices of the resulting particles. Additionally, Rf incorporation plays a vital role; as a water-soluble component, it enhances amorphization and improves wettability. Ultimately, these findings are beneficial for obtaining a rapid onset of action [103], enhancing bioavailability, and avoiding biological and immunological elimination mechanisms.

### 3.11. In Vitro Diffusion

**Figure 13:**
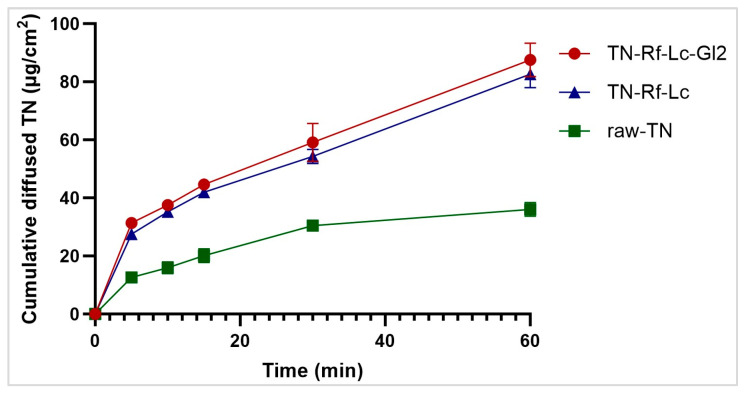
In vitro diffusion profile of raw-TN, TN-Rf-Lc, and TN-Rf-Lc-Gl2 formulations.

An in vitro diffusion test provides a valuable prediction of the drug pharmacokinetics, permeability, and absorption [104]. Via PDDS, reduced doses are delivered due to localized administration and bypassing the hepatic first-pass metabolism; thus, this dose should be diffused to the epithelial cells. The permeation flux rate showed markedly higher TN permeation from TN-Rf-Lc (82.85 ± 4.7 µg/cm^2^/h) and TN-Rf-Lc-Gl2 (87.54 ± 5.7 µg/cm^2^/h) than raw TN (36.05 ± 2.3 µg/cm^2^/h), as shown in Figure 13. This improved in vitro diffusion could be attributed to the consequences of spray-drying and formulation development, namely, particle size micronization, high surface area, less crystalline structure, and enhanced solubility. Both formulations exhibited a more than two-fold increase in permeation (RP_60_: 2.29 for TN-Rf-Lc and 2.43 for TN-Rf-Lc-Gl2). Furthermore, the permeability coefficient K_p_ was found (0.459 and 0.486 cm/h for TN-Rf-Lc and TN-Rf-Lc-Gl2, respectively). Therefore, the developed co-spray-dried fine carriers could be considered an advantage for the local administration of TN for managing asthmatic symptoms. However, the utilized isopropyl myristate created a bio-relevant lipophilic microenvironment for TN partitioning and solubility at the donor–membrane interface [105]. Yet, a cell permeability study is needed to support these results.

A prior study concluded that TN has superior permeability and flux across porcine bronchial cells in comparison to other drugs [106] and exhibited high pH-independent permeability across Caco-2 monolayers, along with total absorption through the human small intestinal epithelium [107,108]. It is considered that TN was classified as a model drug for the validation procedures of permeability investigation methods of other drugs, issued by the World Health Organization (WHO), due to its high permeability [109].

## 4. Discussion

Preliminary experiments of novel combined fine carriers: The initial study investigated the suitability of fine spray-dried carriers composed of Rf and amino acids in binary and ternary combinations. Low concentrations of Lc, Gl, and Ar were screened with a constant Rf base throughout the experimental work. As a result, Ar-containing carriers were found inappropriate due to insufficient in vitro aerodynamic performance due to the hygroscopic properties of Ar, which may lead to quick moisture uptake and increased aggregation possibility. However, Rf-Lc and Rf-Lc-Gl obtained superior morphology, particle size, and aerodynamic performance; hence, they were chosen for further development. Rf and Rf-Gl exhibited uninformative shapes, yet relatively high FPF and acceptable MMAD; therefore, they were also selected for the upcoming formulation.

Findings and their implications of TN co-spray-dried DPIs: The optimization and characterization of TN inhalable powder, utilizing the appropriate Rf-amino acid combinations as fine co-spray-dried carriers, represent significant advancements in PDDS of low-dose TN aimed at enhancing therapeutic outcomes for asthma management. The study demonstrated that co-spray-drying with the utilized excipients promoted the formation of fine spherical particles with enhanced aerodynamic properties, with hollowed structure in TN-Rf-Lc-Gl2, which contains a higher TN quantity. Notably, the application of Lc, either with or without Gl (TN-Rf-Lc and TN-Rf-Lc-Gl2), has the main enhancement effect on morphology and aerodynamic behavior, showing no aggregation with improved aerodynamic and particle size properties (MMAD = 5.33–5.14 µm and FPF = 45.69–47.84%, respectively) compared to SD-TN. On the other hand, TN-Rf and TN-Rf-Gl exhibited particle aggregation proven by SEM images, which indicates less physical stability and contributes to diminishing aerodynamic integrity. Thermal and structural analyses demonstrated a shift towards predominantly amorphous characteristics of post-spray-drying, confirmed by X_c_ values, and DSC and FTIR, supporting the structural transformation. This amorphization shift promotes enhanced in vitro solubility, in vitro TN release rate, and in vitro permeability from TN-Rf-Lc and TN-Rf-Lc-Gl2. Therefore, better-expected bioavailability could be achieved using low-dose TN and minimizing side effects associated with oral and intravenous administration. Additionally, TN-Rf-Lc and TN-Rf-Lc-Gl2 proved enhanced in silico deposition in the bronchial and acinar regions (~40%) with a low exhaled fraction; however, the high extrathoracic fraction (~50%) is a key challenge according to our results and needs to be addressed in further investigations of fine DPIs. Finally, the aerodynamic particle counter assessment, which measured the aerodynamic particle size from different perspectives (number, surface, and volume), confirmed the production of fine microparticles ranging from 1.45 µm to 5.67 µm. These results are considered an added value to the few previous attempts to deliver TN as a DPI applying various excipients, techniques, and even drug combinations [45,47,84]. Moreover, the designed fine carriers could be investigated as a base to formulate different APIs as inhaled powders.

Future directions: A long-term physical stability, or under storage conditions, is highly recommended to support research reliability. However, this work paved the way for further pulmonary applications of various composite fine microparticles. For instance, investigating several fine excipients prepared by different techniques, such as spray-freeze-drying, could provide a broader understanding of the developed DPIs. Eventually, scalability and biological studies should be continued to validate the reproducibility, real efficacy, and safety of such products, and a regulatory framework is also needed. By addressing these limitations, future research could optimize the applicability of fine DPIs.

## 5. Conclusions

Rf combined with low-concentration Lc or Lc-Gl showed promising performance as fine microcarriers of DPIs via spray-drying. TN development as a novel co-spray-dried respirable powder with Rf and amino acid(s) was implemented successfully. TN-Rf-Lc and TN-Rf-Lc-Gl2 exhibited significant enhancement for local respirable application targeting asthma symptoms, optimizing aerodynamic particle size and shape for deep lung administration. TN co-spray-dried samples improved solubility in water and SLF, fast in vitro dissolution in SLF, and enhanced drug diffusion, indicating favorable physicochemical characteristics. Aerodynamic assessment confirmed enhanced deep lung delivery (FPF: 24.97–47.84%; MMAD: 5.14–7.22 µm), with fine microparticles (1.45–5.67 µm). However, Ar-containing carriers were unsuitable due to high aggregation, yielding low FPF (16.31–18.33%). TN-Rf and TN-Rf-Gl were also inconvenient due to high MMAD (6.4–7.2 µm). This approach offers effective TN inhalation treatment, reducing dosing and side effects while improving outcomes.

## Figures and Tables

**Table 1:** The comprehensive composition of the developed carriers and TN samples.

Formulation	TN (%)	Rf (%)	Lc (%)	Gl (%)	Ar (%)
Rf	-	3.5	-	-	-
Rf-Lc	-	3.5	0.75	-	-
Rf-Gl	-	3.5	-	0.75	-
Rf-Ar	-	3.5	-	-	0.75
Rf-Ar-Lc	-	3.5	0.75	-	0.75
Rf-Gl-Lc	-	3.5	0.75	0.75	-
Rf-Gl-Ar	-	3.5	-	0.75	0.75
SD-TN	1.17	-	-	-	-
TN-Rf	1.17	3.5	-	-	-
TN-Rf-Gl	1.17	3.5	-	0.75	-
TN-Rf-Lc	1.17	3.5	0.75	-	-
TN-Rf-Lc-Gl	1.17	3.5	0.75	0.75	-
TN-Rf-Lc-Gl2	1.75	3.5	0.75	0.75	-

**Table 2:** Particle size distribution results of TN spray-dried formulations.

Formulation	D [0.1] * (µm)	D [0.5] * (µm)	D [0.9] * (µm)	Span *
SD-TN	2.10 ± 0.01	4.38 ± 0.05	9.85 ± 1.93	1.77 ± 0.89
TN-Rf	2.19 ± 0.09	4.89 ± 0.19	13.10 ± 3.25	2.21 ± 0.55
TN-Rf-Gl	2.57 ± 0.01	5.96 ± 0.22	16.21 ± 1.70	2.29 ± 0.21
TN-Rf-Lc	1.72 ± 0.04	3.99 ± 0.17	13.19 ± 2.94	2.86 ± 0.63
TN-Rf-Lc-Gl	1.88 ± 0.01	4.43 ± 0.11	19.22 ± 6.63	3.90 ± 1.40
TN-Rf-Lc-Gl2	1.82 ± 0.03	4.23 ± 0.09	13.23 ± 3.47	2.69 ± 0.77

* Results are expressed as average ± SD (n = 3).

**Table 3:** Powder rheology metrics: density and flowability properties of TN spray-dried samples.

Formulation	ρ_bulk_ * (g/cm^3^)	ρ_tapped_ * (g/cm^3^)	HR *	CI * (%)
Raw-TN	1.11 ± 0.03	1.53 ± 0.07	1.37 ± 0.03	27.14 ± 1.34
SD-TN	1.27 ± 0.07	1.75 ± 0.09	1.38 ± 0.01	27.68 ± 0.37
TN-Rf	0.34 ± 0.03	0.45 ± 0.03	1.32 ± 0.04	24.49 ± 2.25
TN-Rf-Gl	0.26 ± 0.04	0.36 ± 0.05	1.40 ± 0.05	28.57 ± 2.78
TN-Rf-Lc	0.36 ± 0.02	0.50 ± 0.03	1.41 ± 0.03	29.24 ± 1.50
TN-Rf-Lc-Gl	0.44 ± 0.02	0.63 ± 0.04	1.43 ± 0.05	30.25 ± 2.36
TN-Rf-Lc-Gl2	0.32 ± 0.04	0.46 ± 0.05	1.45 ± 0.04	31.20 ± 1.88

* Results are expressed as average ± SD (n = 3).

**Table 4:** Aerodynamic characteristics of TN-spray-dried samples.

Formulation	EF * (%)	MMAD * (µm)	FPF * (%)	GSD *	Calculated Delivered Dose (mg)
SD-TN	73.30 ± 1.09	5.35 ± 0.74	32.52 ± 10.63	2.05 ± 0.21	5.75 ± 0.43
TN-Rf	86.92 ± 0.45	6.44 ± 0.08	30.14 ± 0.24	2.71 ± 0.06	5.62 ± 0.10
TN-Rf-Gl	90.26 ± 2.56	7.20 ± 0.95	24.97 ± 4.79	2.37 ± 0.01	7.35 ± 0.79
TN-Rf-Lc	94.40 ± 1.05	5.33 ± 0.82	45.69 ± 4.82	2.68 ± 0.15	9.58 ± 0.80
TN-Rf-Lc-Gl	95.08 ± 3.71	5.61 ± 0.73	38.75 ± 8.02	5.36 ± 0.01	8.26 ± 0.45
TN-Rf-Lc-Gl2	96.45 ± 0.36	5.14 ± 0.00	47.84 ± 1.36	2.72 ± 0.20	8.90 ± 1.44

* Results are expressed as average ± SD (n = 3).

**Table 5:** Aerodynamic particle counter results (number, surface, and volume particle size) of TN-Rf-Lc and TN-Rf-Lc-Gl2.

Parameters	Number Particle Size		Surface Particle Size		Volume Particle Size	
	TN-Rf-Lc	TN-Rf-Lc-Gl2	TN-Rf-Lc	TN-Rf-Lc-Gl2	TN-Rf-Lc	TN-Rf-Lc-Gl2
Median * (µm)	2.01 ± 0.13	1.65 ± 0.10	3.58 ± 0.15	3.49 ± 0.25	4.58 ± 0.09	4.90 ± 0.09
Mean * (µm)	2.30 ± 0.11	2.02 ± 0.13	4.02 ± 0.08	4.02 ± 0.13	5.12 ± 0.17	5.43 ± 0.09
Geometric mean * (µm)	1.97 ± 0.09	1.71 ± 0.10	3.54 ± 0.10	3.41 ± 0.17	4.54 ± 0.10	4.71 ± 0.07
Mode * (µm)	2.49 ± 0.4	1.45 ± 0.14	3.43 ± 0.23	4.33 ± 0.83	4.63 ± 0.15	5.67 ± 0.35
GSD *	1.75 ± 0.02	1.76 ± 0.03	1.67 ± 0.05	1.79 ± 0.05	1.63 ± 0.06	1.73 ± 0.07

* Results are expressed as average ± SD (n = 3).

## Data Availability

The original contributions presented in the study are included in the article; further inquiries can be directed to the corresponding author.

## Supplementary Materials

The following supporting information can be downloaded at https://www.mdpi.com/article/10.3390/pharmaceutics17040466/s1. Figure S1: Calibration curve of TN in distilled water; Figure S2: Calibration curve of TN in SLF; Figure S3: Calibration curve of TN in ethanol 10%; Figure S4: Calibration curve of TN in phosphate buffer pH = 7.4.

## Abbreviations

ACI	Andersen Cascade Impactor
APC	Aerodynamic Particle Counter
API	Active Pharmaceutical Ingredient
APSD	Aerodynamic Particle Size Distribution
CI	Carr’s Index
DPI	Dry Powder Inhaler
DSC	Differential Scanning Calorimetry
EF	Emitted Fraction
FPF	Fine Particle Fraction
FTIR	Fourier-Transform Infrared
HR	Hausner Ratio
MMAD	Median Mass Aerodynamic Diameter
PDDS	Pulmonary Drug Delivery System
PSD	Particle Size Distribution
SD	Standard Deviation
SEM	Scanning Electron Microscopy
SD-TN	Spray Dried Theophylline
TGA	Thermogravimetric Analysis
TN	Theophylline Anhydrous
X_c_	Crystallinity Index
XRPD	X-Ray Powder Diffraction
