# Supplementary material for: Optimization, In Vitro, and In Silico Characterization of Theophylline Inhalable Powder Using Raffinose-Amino Acid Combination as Fine Co-Spray-Dried Carriers
Source: Pharmaceutics. 2025 Apr 3;17(4):466. doi: 10.3390/pharmaceutics17040466 (PMC12030175; doi:10.3390/pharmaceutics17040466)
Supplement: Supplementary file 1 [file pharmaceutics-17-00466-s001.zip › pharmaceutics-3549292-supplementary.pdf]

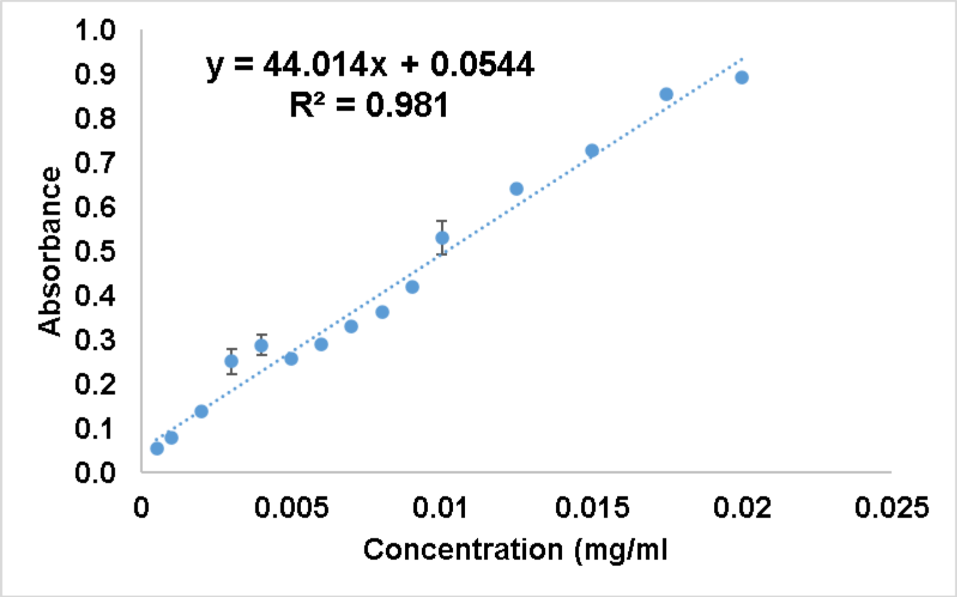

Figure S1. Calibration curve of TN in distilled water.

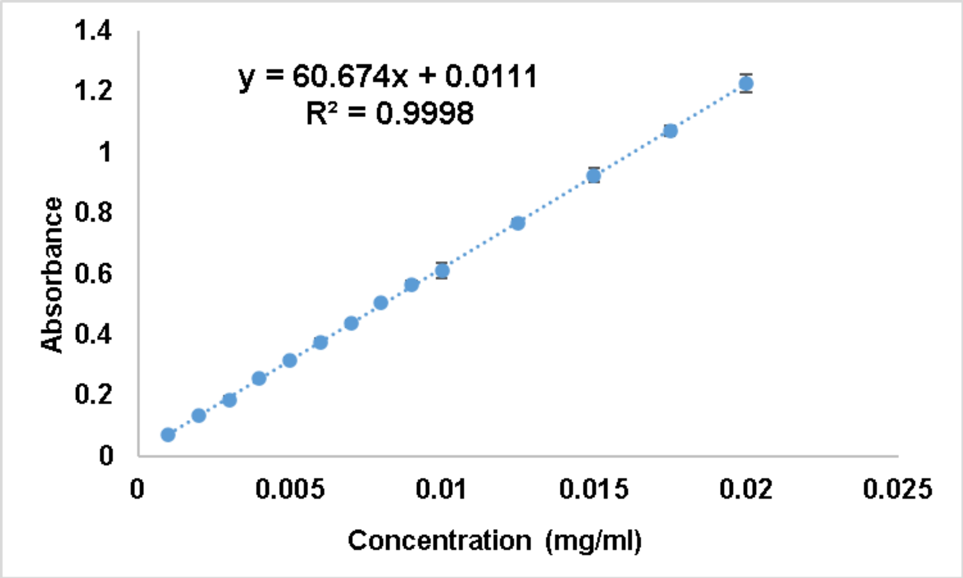

Figure S2. Calibration curve of TN in SLF.

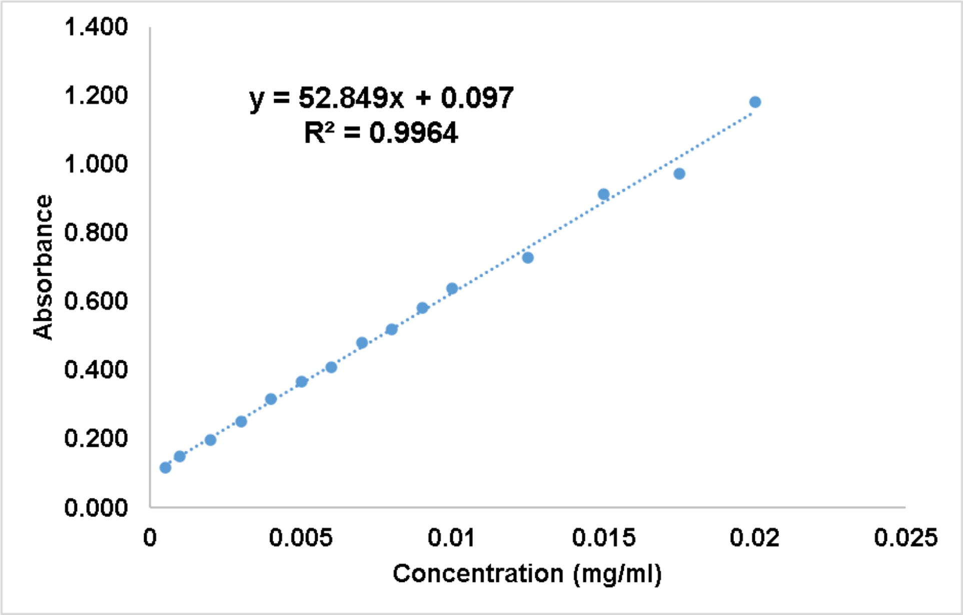

Figure S3. Calibration curve of TN in ethanol 10%.

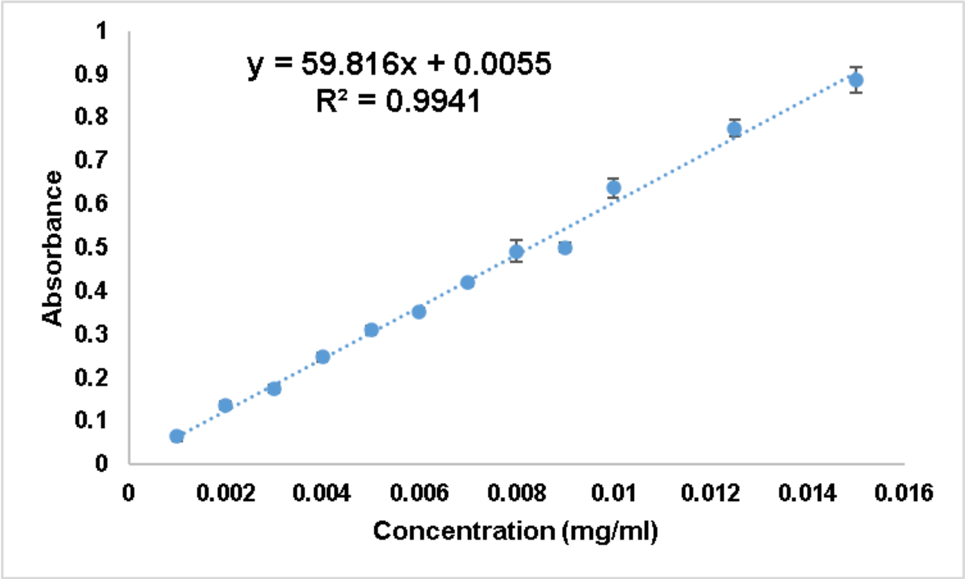

Figure S4. Calibration curve of TN in phosphate buffer pH=7.4.
